# Anti-steatotic effect of *Opuntia ficus-indica* extracts rich in betalains and phenolics from fruit peel and pulp of different varieties in in vitro models

**DOI:** 10.1007/s13105-025-01097-4

**Published:** 2025-06-20

**Authors:** Irene Besné-Eseverri, Jenifer Trepiana, Itziar Eseberri, Andrea Gómez-Maqueo, M. Pilar Cano, Joao Tomé-Carneiro, Alberto Dávalos, María P. Portillo

**Affiliations:** 1 Nutrition and Obesity Group, Department of Nutrition and Food Science, Faculty of Pharmacy, University of the Basque Country (UPV/EHU), Lucio Lascaray Research Centre, Vitoria-Gasteiz, Spain; 2 CIBERobn Physiopathology of Obesity and Nutrition, Institute of Health Carlos III (ISCIII), Madrid, Spain; 3 Bioaraba Health Research Institute, Vitoria-Gasteiz, Spain; 4 Laboratory of Phytochemistry and Plant Food Functionality, Biotechnology and Food Microbiology Department, Institute of Food Science Research (CIAL) (CSIC-UAM), Madrid, Spain; 5 Laboratory of Functional Foods, IMDEA Food, CEI UAM+CSIC, Carretera de Cantoblanco, 8, Madrid, 28049 Spain; 6 Laboratory of Epigenetics of Lipid Metabolism, IMDEA Food, CEI UAM+CSIC, Carretera de Cantoblanco, 8, Madrid, 28049 Spain

**Keywords:** *Opuntia ficus-indica*, Fruit extracts, AML12 hepatocytes, Organoids, MAFLD

## Abstract

*Opuntia ficus-indica* exhibits antioxidant, anti-inflammatory and anti-hyperglycemic properties, making it a promising candidate for the prevention and treatment of metabolic dysfunction-associated fatty liver disease. However, its effects on triglyceride accumulation remain largely unexplored. The aim of the present study is to evaluate the anti-steatotic effect of peel and pulp extracts from different varieties of *Opuntia ficus-indica* fruits (*Pelota*, *Colorada* and *Sanguinos*) in hepatic murine in vitro models, using both AML12 hepatocytes and hepatic organoids. The pulp extracts of *Pelota* and *Colorada* varieties, as well as both peel and pulp extracts of *Sanguinos*, were effective in reducing palmitic acid-induced triglyceride accumulation in AML12 hepatocytes. The doses that caused the greatest triglyceride reduction were 50 µg/mL of the pulp of *Pelota* and 100 µg/mL for the other extracts. The potential mechanisms underlying these effects seem to be associated, at least in part, with the inhibition of fatty acid uptake and triglyceride assembly. The pulp extract of the *Colorada* variety was able to prevent triglyceride accumulation also in hepatic organoids, likely due to downregulation of fatty acid transporters. These findings underscore the value of employing diverse in vitro models (e.g., 2D, 3D) to investigate the potential effects of these extracts, and suggest that the pulp extract of the *Colorada* variety may be effective in preventing steatosis.

## Key points

● Opuntia ficus-indica extracts prevent triglyceride content in in vitro hepatic models.

● Pulp O. ficus-indica var. Colorada reduced triglyceride accumulation in both models.

● The anti-steatotic effect may be due to a downregulation in fatty acid uptake.

## Introduction

Metabolic-associated fatty liver disease (MAFLD) is the most common chronic liver disease. It encompasses stages of varying severity, ranging from relatively benign steatosis to more advanced conditions, including steatohepatitis, cirrhosis and hepatocellular carcinoma [1]. This term was proposed as a more precise definition of non-alcoholic fatty liver disease (NAFLD) [2], since it emphasises metabolic dysfunction as a stronger indicator of fatty liver-associated risks [3]. An overall prevalence of 33% has been estimated for MAFLD, with significant variation depending on region and age [1].

Lifestyle modifications such as dietary adjustments and exercise training, are the preferred preventive and therapeutic strategies. Nevertheless, the results are not consistently optimal [4]. In this context, bioactive compounds present in plant-based foods could serve as a potential complementary tool for this approach. *Opuntia* cactus (from the Cactaceae family) is an important source of bioactive compounds that include carotenoids, amino acids, vitamins, fibres, betalains (betacyanins and betaxanthins) and phenolic compounds [5–7]. Also, *Opuntia* polysaccharides, present in cladodes and fruits, are of particular interest due to their high content of pectic substances and mucilage, which exhibit various biological activities depending on their composition [8, 9].

Several antioxidant, anti-inflammatory and anti-hyperglycemic activities of *Opuntia ficus-indica* extracts, which are closely associated with MAFLD, have been reported [10]. However, data concerning their ability to reduce the accumulation of hepatic triglycerides are scarce [11–13].

In this scenario, the interest of the present study focuses on the anti-steatotic effect of *Opuntia ficus-indica* L. extracts, rich in betalains and phenolic compounds, derived from the peel or pulp of three varieties (*Pelota*, *Colorada* and *Sanguinos*). For this purpose, a screening experiment was conducted in murine hepatocyte cultures. Additionally, the primary mechanisms underlying the observed effects were explored. Although traditional two-dimensional (2D) cultures are the most commonly used in vitro model, they have certain limitations, and complementary (more complex) models should be tested whenever possible. These include organoids, which are three-dimensional (3D) mini versions of organs or tissues derived from stem cells that can self-organize and differentiate into structures that replicate the morphology and functions of their in vivo counterparts [14]. Thus, organoids have the potential to contribute to a better understanding of the mechanisms behind certain diseases and treatments. Taking this into account, the present study also investigates murine liver organoids.

## Materials and methods

### Reagents

For AML12 hepatocytes, Dulbecco modified Eagle’s minimal essential medium (DMEM)/HAM’s F12 (F-12 Nutrient medium) Glutamax, insulin, transferrin, and selenium (ITS) were obtained from Thermofisher (Waltham, MA, USA). Foetal bovine serum (FBS) was purchased from Corning (New York, NY, USA). Streptomycin-penicillin solution and trypsin/EDTA were sourced from Lonza (Basel, Switzerland), and palmitic acid (PA) from Sigma-Aldrich (St Louis, MO, USA). Regarding hepatic organoids, Hepaticult medium and other reagents were purchased from STEMCELL technologies (Grenoble, France), unless stated otherwise. Matrigel was obtained from Corning (New York, NY, USA).

### *Opuntia ficus-indica* fruit extracts

**Fig. 1:**
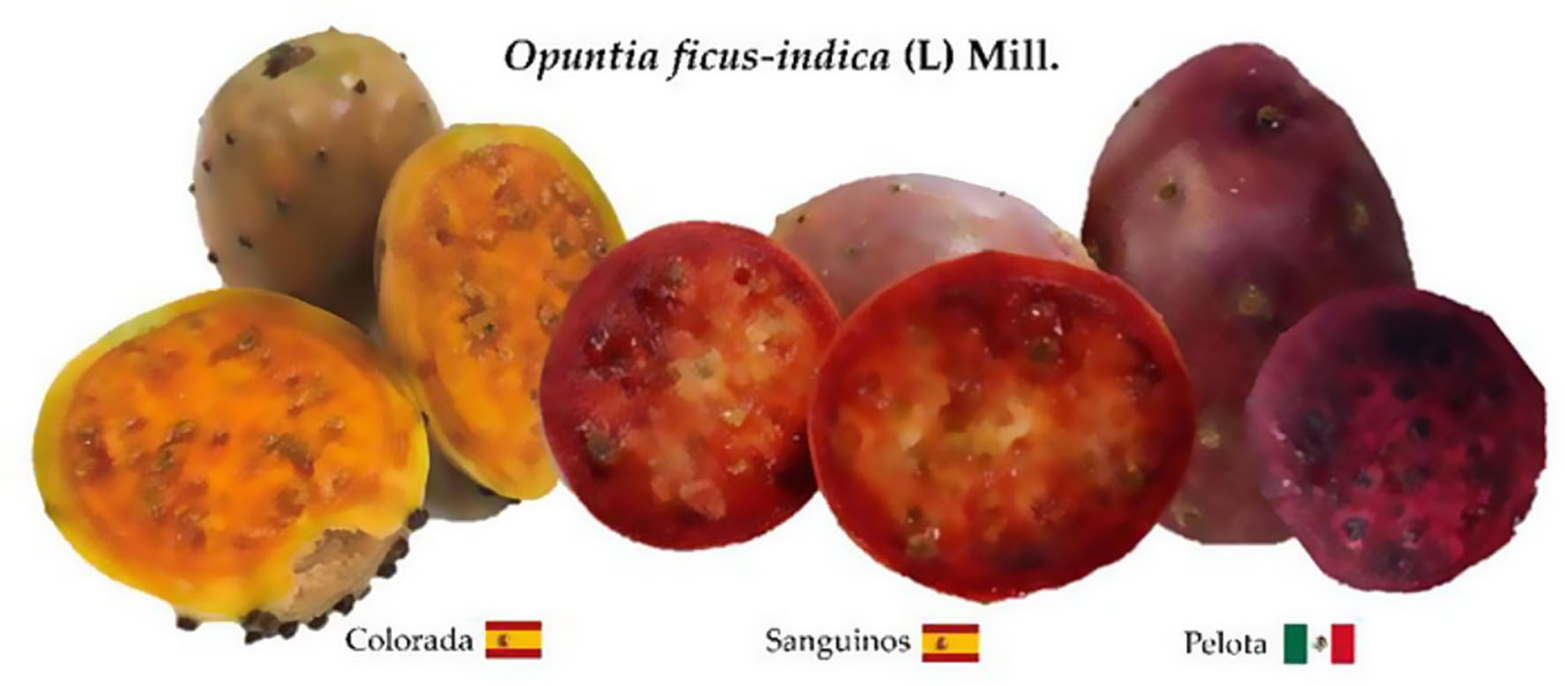
Fruits of the *Opuntia ficus-indica* varieties: *Colorada*, *Sanguinos* and *Pelota*

Spanish orange var. *Colorada* prickly pears were collected from Fasnia (Tenerife, Canary Islands, Spain; 28°2′ N, 16°4′ W; 446 metres above sea level), Spanish red *Sanguinos* prickly pears from Bioarchen in Archena (Murcia, Spain; 38°7′ N, 1°180′ W; 121 m. a. s. l.), and purple Mexican *Pelota* prickly pears were provided by Agroproductores La Flor de Villanueva in San Sebastián Villanueva Acatzingo (Puebla, Mexico; 19°1’ N, 97°4′ W; 121 m. a. s. l.) (Fig. 1).

The fruits were washed and selected based on uniform maturity, size, and absence of defects, then separated into peels and pulps. The tissues were cut into small pieces (20 × 20 mm), vacuum-sealed in polyethylene bags and frozen using liquid nitrogen. Following seed removal, samples were freeze-dried for five days at − 45 °C and 1.3 × 10 − 3 MPa (LyoBeta 15, Azbil Telstar, S.L., Terrasa, Spain), and pulverised (Grindomix GM200, Retsch, Germany) to a fine particle size (< 2 mm). Aqueous prickly pear extracts were obtained from freeze-dried tissues by repeated extraction with methanol: water (1:1, v: v) and methanol to obtain extracts rich in betalains and phenolic compounds [15]. Aliquots of each extract were freeze-dried and stored at − 20 °C until they were required for high performance liquid chromatography (HPLC) analysis and in vitro experiments.

### Bioactive compound quantification by HPLC

Betalains and phenolic compounds in prickly pear fruit extracts were simultaneously determined by HPLC [15, 16]. A 1200 Series Agilent HPLC System (Agilent Technologies, Santa Clara, CA, USA) with a reverse-phase C18 column (Zorbax SB-C18, 250 × 4.6 mm i.d., S-5 μm; Agilent) was used at 25 °C. Betalains and phenolic compounds were identified according to their retention times, UV/Vis and mass spectral data were compared to those of commercial, semi-synthesised or purified standards. Quantitation of betanin, indicaxanthin, piscidic acid, and isorhamnetin glycosides was conducted using calibration curves for each of the corresponding isolated and semi-synthetised standards, with five points in a range of 0–300 µg/mL. The content of each compound was expressed in mg/g dry weight (Table 1).

**Table 1:** Primary individual betalain and phenolic compound content (mg/g dry weight) in prickly pear (*Opuntia ficus-indica*) peel and pulp extracts [15, 16]

*Opuntia ficus-indica* extracts						
	*Pelota*		*Sanguinos*		*Colorada*	
Compound	Peel	Pulp	Peel	Pulp	Peel	Pulp
Indicaxanthin	0.06 ± 0.00	0.23 ± 0.01	0.09 ± 0.00	0.12 ± 0.01	0.45 ± 0.02	0.74 ± 0.04
Betanin	1.88 ± 0.09	2.79 ± 0.14	0.80 ± 0.04	0.27 ± 0.01	0.09 ± 0.00	0.08 ± 0.00
Piscidic acid	68.79 ± 3.44	4.19 ± 0.21	52.56 ± 2.63	6.46 ± 0.32	50.99 ± 2.55	5.79 ± 0.29
IG1	0.59 ± 0.03	n.d.	0.34 ± 0.02	n.d.	0.22 ± 0.01	n.d.
IG2	0.10 ± 0.03	n.d.	0.29 ± 0.01	n.d.	0.21 ± 0.01	n.d.
IG4	0.05 ± 0.00	n.d.	0.22 ± 0.01	n.d.	0.12 ± 0.01	n.d.
IG5	0.38 ± 0.02	n.d.	0.46 ± 0.02	n.d.	0.32 ± 0.02	n.d.

Data are presented as means ± SEM (standard error of the mean) of three independent experiments conducted in sextuplicate. IG1: isorhamnetin glycosyl-rhamnosyl-rhamnoside, IG2: isorhamnetin glucosyl-rhamnosyl-pentoside, IG4: isorhamnetin glucosyl-pentoside, IG5: isorhamnetin glucosyl-rhamnoside. n.d.: not detected

### Mouse AML12 hepatocytes

#### Culture and maintenance

AML12 mouse hepatocytes (alpha mouse liver 12; ATCC^®^ CRL-2254™) were obtained from ATCC (Manassas, VA, USA). These cells were cultured in 75 cm^2^ flasks in DMEM/HAM’s F12 Glutamax, supplemented with 10% heat inactivated foetal bovine serum, 5 µg/mL insulin, 5 µg/mL transferrin, 5 ng/mL selenium, 40 ng/mL dexamethasone and 1% penicillin/streptomycin (10,000 U/mL). AML12 cells were grown at 37 °C in a humidified atmosphere with 5% CO_2_. When the cell monolayer reached 75% confluence, the cells were detached using a trypsin-EDTA solution and harvested for subsequent experiments.

#### Experimental design

An in vitro model mimicking the hepatocyte condition in fatty liver was created using mouse AML-12 hepatocytes, cultured in 6-well plates and incubated with 0.3 mM of PA for 18 h to induce triglyceride accumulation, according to previous studies [17–19]. In the first experiment, hepatocytes were co-incubated with PA and the *Opuntia ficus-indica* extracts at concentrations of 10, 25, 50, 100–200 µg/mL (diluted in 95% water) for 18 h. Control cells were treated with the same volume of the vehicle. All the experiments were performed at least three times.

### Optical microscopy analysis of steatotic AML12 hepatocytes

The accumulation of lipid droplets was examined using optical microscopy. Hepatocytes plated in 6-well culture plates and treated for 18 h were imaged using an Olympus CH optical microscope (Olympus, Tokyo, Japan), and analysed at a 40 x magnification. Cellular characteristics were assessed using ImageJ software (NIH, Bethesda, Maryland, MD, USA).

### Cell viability assay

Live cell number was determined using the crystal violet assay, based on cell staining with crystal violet [20]. Briefly, hepatocytes were seeded in 96-well tissue culture plates at 5 × 10^3^ cells per well, and three days after plating, the cells were treated with the pertinent compounds for 18 h. After treatments, cells were washed with phosphate-buffered saline (PBS), fixed in 3.7% formaldehyde, and stained with 0.25% crystal violet for 20 min in the dark. The resulting crystals were then solubilised with 33% acetic acid and absorbance was registered at 590 nm in an iMark microplate reader (Bio-Rad, Hercules, CA, USA). Cell viability was expressed as a percentage of control cells.

### Determination of triglyceride levels

At the end of each treatment, the cell medium was discarded, and the cells were utilised for triglyceride measurement. Hepatocytes were washed extensively with PBS, and the cell suspension was sonicated on ice in 10 mM Tris-HCl pH 7.4, 150 mM NaCl and 1 mM EDTA, with five 5-s bursts using a Branson Sonifier SFX550 (San Luis, Missouri, MO, USA) fitted with a microtip. Triglyceride content was then measured with a commercial kit (Spinreact, Girona, Spain). Protein measurements were performed using the Bradford method [21]. Triglyceride content values were obtained as mg triglycerides/mg protein and expressed as a percentage of control cells.

### Protein immunodetection

Phospho-acetyl-CoA carboxylase (pACC), total acetyl-CoA carboxylase (ACC), fatty acid synthase (FAS), carnitine palmitoyltransferase 1 A (CPT1A), CD36 molecule (CD36), fatty acid transporter protein 2 (FATP2), diacylglycerol *O*-acyltransferase 2 (DGAT2), and glyceraldehyde 3-phosphate dehydrogenase (GAPDH) expression were determined by Western Blot analysis.

Samples treated with *Opuntia ficus-indica* extracts resulting in the highest reduction in triglyceride content were utilized for this assay, i.e., pulp of var. *Pelota* at 50 µg/mL (PPu50), pulp of var. *Colorada* at 100 µg/mL (CPu100), peel of var. *Sanguinos* at 100 µg/mL (SPe100) and pulp of var. *Sanguinos* at 100 µg/mL (SPu100). Cellular protein extracts were denatured at 95 °C for five minutes in Laemmli buffer [22] and subsequently separated by electrophoresis employing 4–15% SDS-polyacrylamide gels. The proteins were transferred to polyvinylidene difluoride (PVDF) membranes via electroblotting at a constant amperage (1 mA/cm^2^). Membranes were blocked at room temperature for 1.5 h, with 4% BSA, and incubated overnight at 4 °C with the following primary antibodies: pACC and ACC (1:1000; Cell Signaling, Danvers, MA, USA), CD36 (1:1000; Cell Signaling, Danvers, MA, USA), CPT1A (1:1000; Abcam, Cambridge, UK), FATP2 (1:1000; Santa Cruz Biotech, Dallas, TX, USA), DGAT2 (1:1000; Abcam, Cambridge, UK) and GAPDH (1:1000; Abcam, Cambridge, UK). After washing, membranes were exposed for two hours at room temperature to polyclonal anti-mouse (1:5000) (Santa Cruz Biotech, Dallas, TX, USA) for CPT1A and GAPDH, anti-rabbit (1:5000) for pACC, ACC and CD36, and anti-goat (1:5000) for FATP2 and DGAT2. The bound antibodies were detected using an ECL system (Thermo Fisher Scientific Inc., Rockford, IL, USA) and the blots were imaged with a ChemiDoc™ MP Imaging System (Bio-Rad, Hercules, CA, USA). GAPDH or the corresponding phosphorylated forms served as the loading control.

### Mouse hepatic organoids

#### Culture and differentiation

All procedures were conducted in accordance with the guidelines of Directive 2010/63/UE. Male C57BL/6 mice were purchased from Charles River (Écully, France) and allowed a 7-day acclimatisation period prior to use. After euthanasia, mice livers were cut into 3–5 mm pieces and digested following Stemcell’s protocol as previously reported [23]. Briefly, samples were submitted to sequential 20 min incubations at 37 °C with a digestion solution (containing dispase (1 U/mL) and collagenase IV (1 mg/mL)) until all pieces were digested. The resulting solution was passed through 70 μm filter and subsequently through a reversible 37 μm strainer to recover the ductal fragments. Fragments were resuspended in matrigel and 30 µL domes were pipetted on the wells of a pre-warmed 24-well plate. After solidification, Hepaticult was added and this medium was changed every 2–3 days, and organoids were passaged after 7 days. When organoids were available in sufficient amounts (usually after 2–3 passages), they were seeded in 48-well plates with Hepaticult. On day 3 after seeding, Hepaticult medium was changed to differentiation medium as defined by Broutier et al., (2016) [24], replacing medium every 2–3 days until a total of 9 days was reached. Finally, differentiation medium containing 3 µM dexamethasone was provided for 3 days (with daily changes) to conclude organoid differentiation procedure.

### Experimental design

Organoids were exposed to 0.3 mM of palmitic acid (to induce triglyceride accumulation) either alone or in combination with the *Opuntia ficus-indica* treatments that were shown to reduce maximum triglycerides levels in the previous study conducted with AML12 hepatocytes. Hence, pulp extract of *Colorada* variety at 100 µg/mL (CPu100), peel extract of *Sanguinos* variety at 100 µg/mL (SPe100) and pulp extract of *Sanguinos* variety at 100 µg/mL (SPu100) were supplemented to organoids, for 18 h. Control groups received an equal volume of vehicle. Each experiment was performed in triplicate. After 18 h of exposure, wells were washed with PBS, and domes were collected in TRIzol^®^ reagent for transcriptomic analysis or in PBS for triglyceride quantification. For triglyceride measurement, domes were resuspended in PBS, centrifuged at 290 g to remove Matrigel, and then organoids were resuspended with 150 µl of PBS. Samples were ultrasonicated as described for AML12 hepatocytes and immediately stored at − 80 °C until quantification.

### Determination of triglyceride levels

The same method described above for AML12 hepatocytes was used for triglyceride determination in hepatic organoids. Triglyceride levels were represented as mg triglycerides/mg protein and expressed as a percentage of control cells.

### RNA extraction and RT-qPCR analysis

Due to the small sample volume obtained from organoids, gene expression (PCR analysis) was measured instead of protein expression (western blot) to determine the mechanisms of action. Total RNA was extracted from organoids using RNeasy Mini Spin Columns (Qiagen, Spain) according to the manufacturer’s instructions and quantified using a Nanodrop 2000. For mRNA analysis, 100 ng of RNA was converted to cDNA using PrimeScript™ RT Reagent Kit (Takara, Japan). Quantitative real-time PCR was conducted using the Mir-X™ miRNA qRT-PCR TB Green^®^ Kit (Takara, Japan) on a QuantStudio™ 12 K Flex Real-time PCR system (Applied Biosystems). The sequences of quantitative PCR primers are listed in Table 2. PCR conditions included 10s at 95 °C, followed by 30s at 60 °C and 30s at 95 °C. Dissociation curves were performed as follows: 60s at 95 °C, followed by denaturation at 95 °C for 5s, and annealing and extension at 60 °C, for 20s. Gene expression levels were calculated using the 2^−ΔΔCt^ relative quantification method [25], with normalisation to RN18S, β-actin and RPLP0 expression.

**Table 2:** Primer sequences for quantitative Real-Time PCR amplification

SYBR Green RT-PCR		
Gene	Sense Primer 5´- 3´	Antisense Primer 5´- 3´
*Acc*	GGA CCA CTG CAT GGA ATG TTA	TGA GTG ACT GCC GAA ACA TCT
*Cd36*	TTG AAA AGT CTC GGA CAT TG	TCA GAT CCG AAC ACA GCG TA
*Cpt1a*	CCA AGT ATC TGG CAG TCG A	CGC CAC AGG ACA CAT AGT
*Dgat2*	GCG CTA CTT CCG AGA CTA CT	GGG CCT TAT GCC AGG AAA CT
*Fabp1*	ACC TCA TCC AGA AAG GGA AGG ACA	TTGGGTCCATAGGTGATGGTGAGT
*Fasn*	TCC TTT GAT GAT TCA GGG AG	TAC AGA GAG CAG ATG AGT TG
*Fatp2*	GGT ATG GGA CAG GCC TTG CT	GGG CAT TGT GGT ATA GAT GA

*Acc*: acetyl-CoA carboxylase; *Cd36*: CD36 molecule; *Cpt1a*: carnitine palmitoyltransferase 1 A; *Dgat2*: diacylglycerol *O*-acyltransferase 2; *Fabp1*: fatty acid binding protein 1; *Fasn*: fatty acid synthase; *Fatp2*: fatty acid transport protein 2

### Statistical analysis

Data were presented as mean ± standard error of the mean (SEM) derived from three independent experiments. Statistical analysis was conducted using SPSS 24.0 (SPSS Inc., Chicago, IL, USA). Following the verification of normal distribution of the variables through the Shapiro-Wilks normality test, data were analysed using one-way ANOVA, followed by the Newman-Keuls post hoc test, to compare different doses of each *Opuntia ficus-indica* extract with the PA-treated and control cells. The same method was used to assess the statistical differences among the treatments that resulted in the greatest triglyceride decrease. Statistical significance was determined at *p* < 0.05.

## Results

### Cell viability in AML12 hepatocytes treated with *Opuntia ficus-indica* fruit extracts

**Fig. 2:**
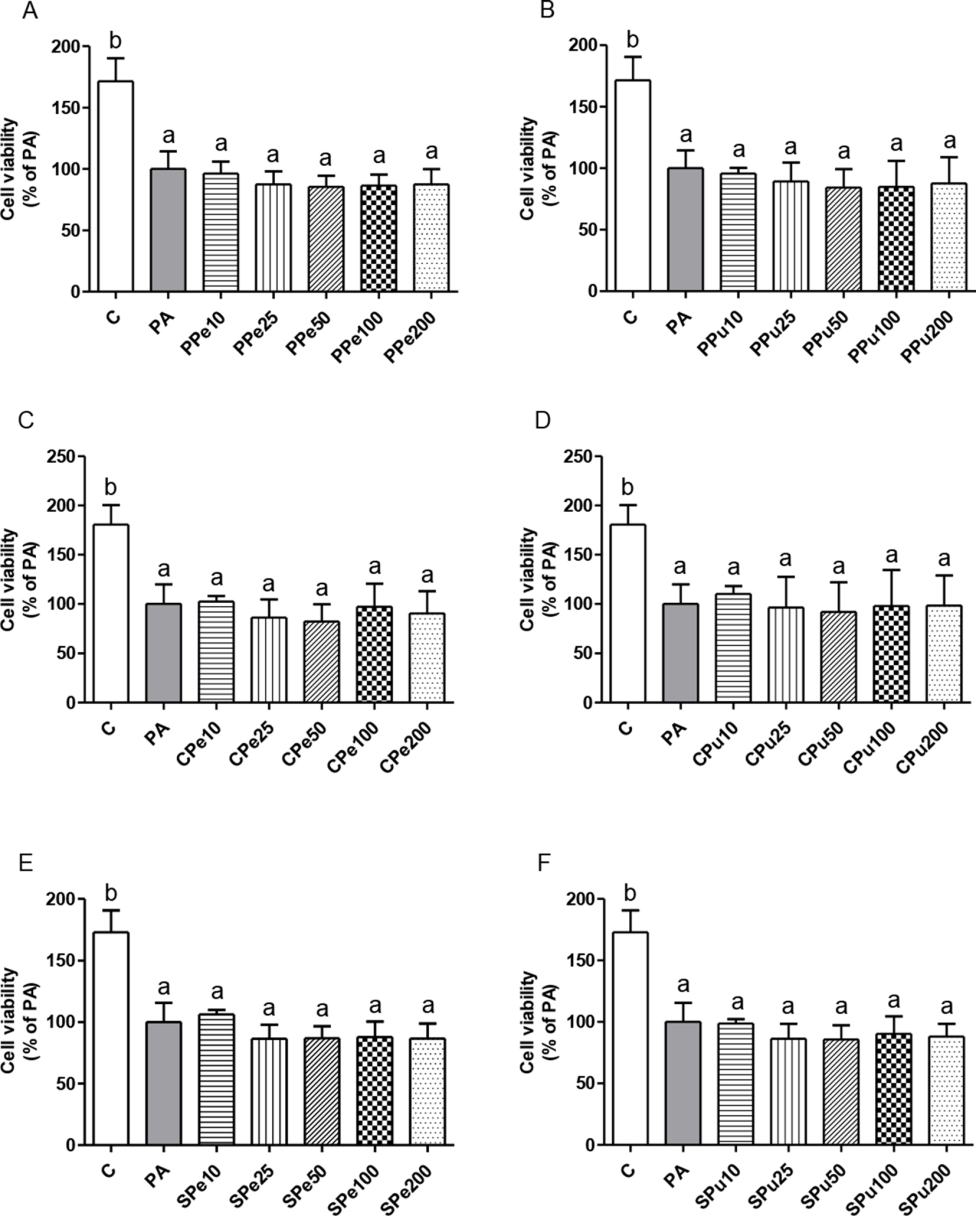
Cell viability in AML12 hepatocytes treated with 0.3 mM palmitic acid (PA) in the presence or absence of *Opuntia ficus-indica* extracts: *Pelota* peel (PPe, A) and pulp (PPu, B), *Colorada* peel (CPe, C) and pulp (CPu, D), and *Sanguinos* peel (SPe, E) and pulp (SPu, F) at concentrations of 10 µg/mL, 25 µg/mL, 50 µg/mL, 100 µg/mL and 200 µg/mL for 18 h. Data are presented as means ± SEM. Differences among groups for each extract were determined using one-way ANOVA followed by the Newman-Keuls post hoc test. Values not sharing a common letter are significantly different (*p* < 0.05)

The viability of AML12 hepatocytes was evaluated following treatment with *Opuntia ficus-indica* extracts to confirm that none of the treatments induced cell damage or toxicity. The incubation of AML12 hepatocytes with PA alone reduced cell viability (-38%) compared to the control group. When *Opuntia ficus-indica* extracts and PA were added simultaneously to AML12 hepatocytes, there was no effect in viability beyond what was observed after the exposure to PA alone, confirming that none of the treatments induced further cell damage or toxicity in the whole range of tested concentrations (Figs. 2A − F).

### Triglyceride levels in hepatocytes treated with *Opuntia ficus-indica* fruit extracts

**Fig. 3:**
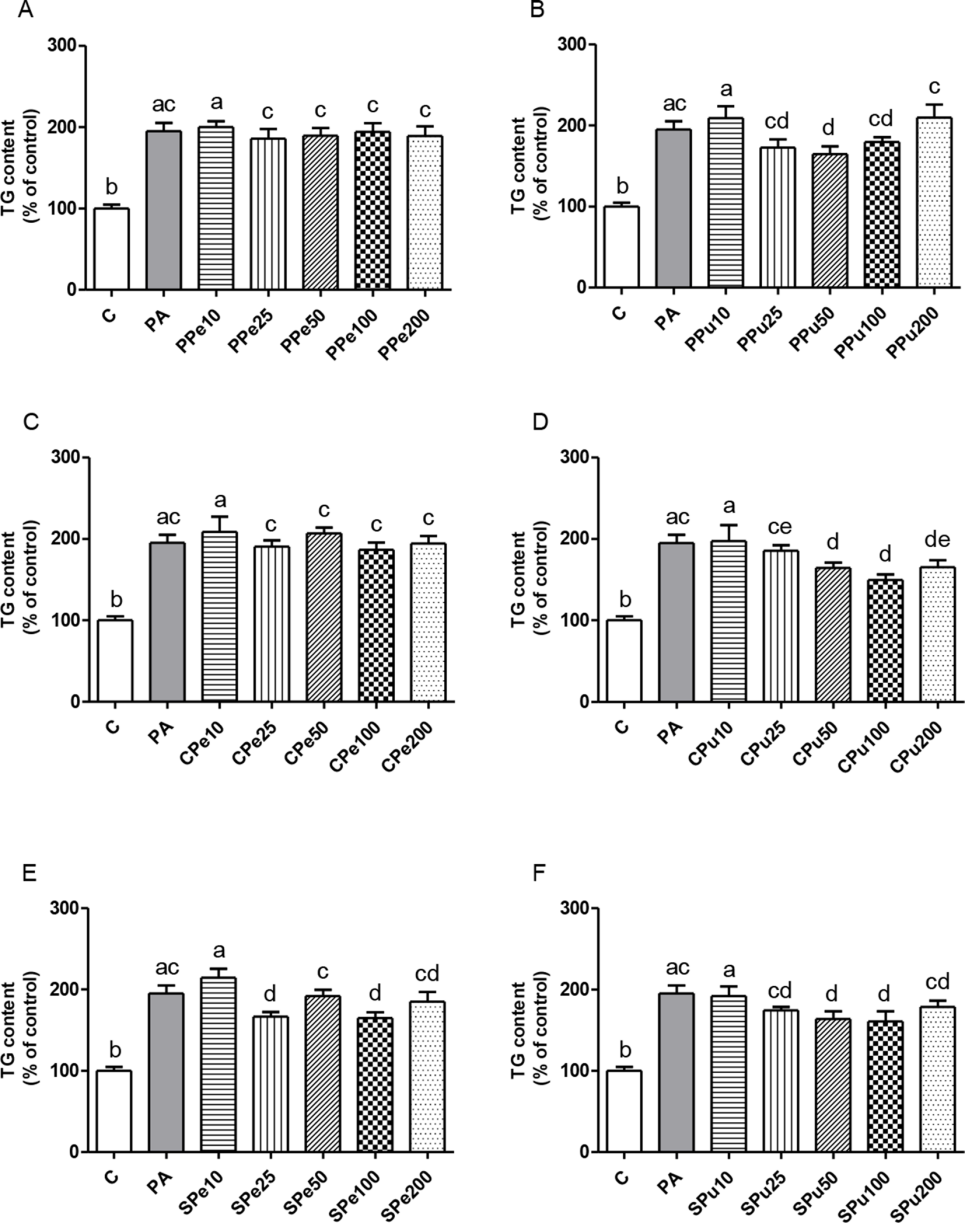
Triglyceride (TG) content in AML12 hepatocytes treated with 0.3 mM palmitic acid (PA) in the presence or absence of *Opuntia ficus-indica* extracts: *Pelota* peel (PPe, A) and pulp (PPu, B), *Colorada* peel (CPe, C) and pulp (CPu, D), and *Sanguinos* peel (SPe, E) and pulp (SPu, F) at concentrations of 10 µg/mL, 25 µg/mL, 50 µg/mL, 100 µg/mL or 200 µg/mL for 18 h. Data are presented as means ± SEM. Differences among groups were determined using one-way ANOVA followed by the New-man-Keuls post hoc test. Values not sharing a common letter are significantly different (*p* < 0.05)

Statistical differences were shown between the PA group and the control cells (*p* < 0.05) in all cases. As shown in Figs. 3A − F, incubation of hepatocytes with *Opuntia ficus-indica* var. *Pelota* extracts (peel or pulp) did not alter triglyceride accumulation, with the exception of the pulp extract at a dose of 50 µg/mL in comparison with the PA group. Concerning *Opuntia ficus-indica* var. *Colorada*, significant reductions were observed with the pulp extract at doses of 50, 100 and 200 µg/mL, whereas the peel extract showed no effect. Lastly, with regard to *Opuntia ficus-indica* var. *Sanguinos*, the peel extract exhibited a significant anti-steatotic effect at concentrations of 25 and 100 µg/mL, while the pulp extract was effective at 50 and 100 µg/mL. Overall, a dose-response patterns were not observed. For both extracts (*Colorada* and *Sanguinos*), no significant differences were found between the effective doses.

In order to determine the most effective treatment in decreasing triglyceride levels, the extracts and concentrations that induced the highest percentages of triglyceride content reduction vs. PA-treated cells (Table 3) were compared. No significant differences were found among all the treatments showing the highest triglyceride reduction capability, i.e., the *Pelota* variety pulp extract at 50 µg/mL, the *Colorada* variety pulp extract at 100 µg/mL, and the peel and pulp extracts of the *Sanguinos* variety at 100 µg/mL, in order to determine the most effective treatment. These findings suggest that all extracts at the doses yielding maximum triglyceride reduction are similarly efficient.

**Table 3:** Triglyceride content reduction (expressed as a percentage) in AML12 hepatocytes, with regard to PA hepatocyte content, induced by the effective *Opuntia ficus-indica* extracts

Extracts	Triglyceride content reduction (%)
*Pelota* pulp (50 µg/mL)	16.3 ± 4.5
*Colorada* pulp (50 µg/mL)	13.2 ± 10.3
*Colorada* pulp (100 µg/mL)	22.4 ± 3.6
*Colorada* pulp (200 µg/mL)	12.4 ± 10.6
*Sanguinos* peel (25 µg/mL)	11.3 ± 10.8
*Sanguinos* peel (100 µg/mL)	14.0 ± 3.7
*Sanguinos* pulp (50 µg/mL)	11.3 ± 11.9
*Sanguinos* pulp (100 µg/mL)	17.2 ± 10.4

### Optical microscopy analysis of steatotic AML12 hepatocytes

**Fig. 4:**
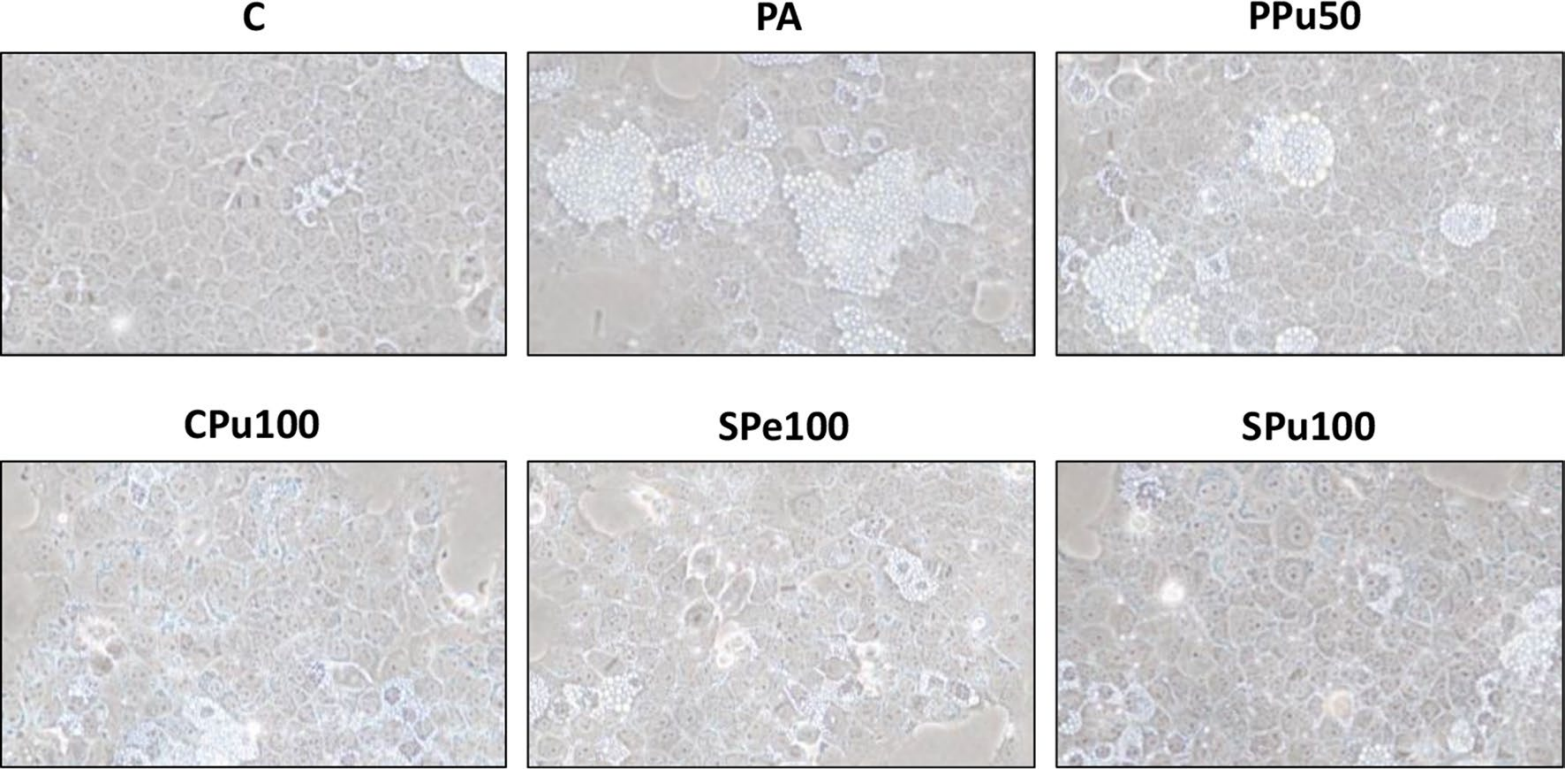
Optical microscopy images (taken with a 40x objective) showing structural features in AML12 hepatocytes treated with 0.3 mM palmitic acid (PA) in the presence or absence of *Opuntia ficus-indica* extracts: *Pelota* pulp (PPu, 50 µg/mL), *Colorada* pulp (CPu; 100 µg/mL), *Sanguinos* peel (SPe, 100 µg/mL) and pulp (SPu, 100 µg/mL) for 18 h

The optical microscopy analysis revealed that the treatment with PA alone induced steatosis in AML12 hepatocytes, while cells co-incubated with the most effective concentrations of *Opuntia ficus-indica* extracts showed smaller dispersed lipid droplets in the cytoplasm (Fig. 4).

### Effect of *Opuntia ficus-indica* fruit extracts on proteins related to triglyceride metabolism

**Fig. 5:**
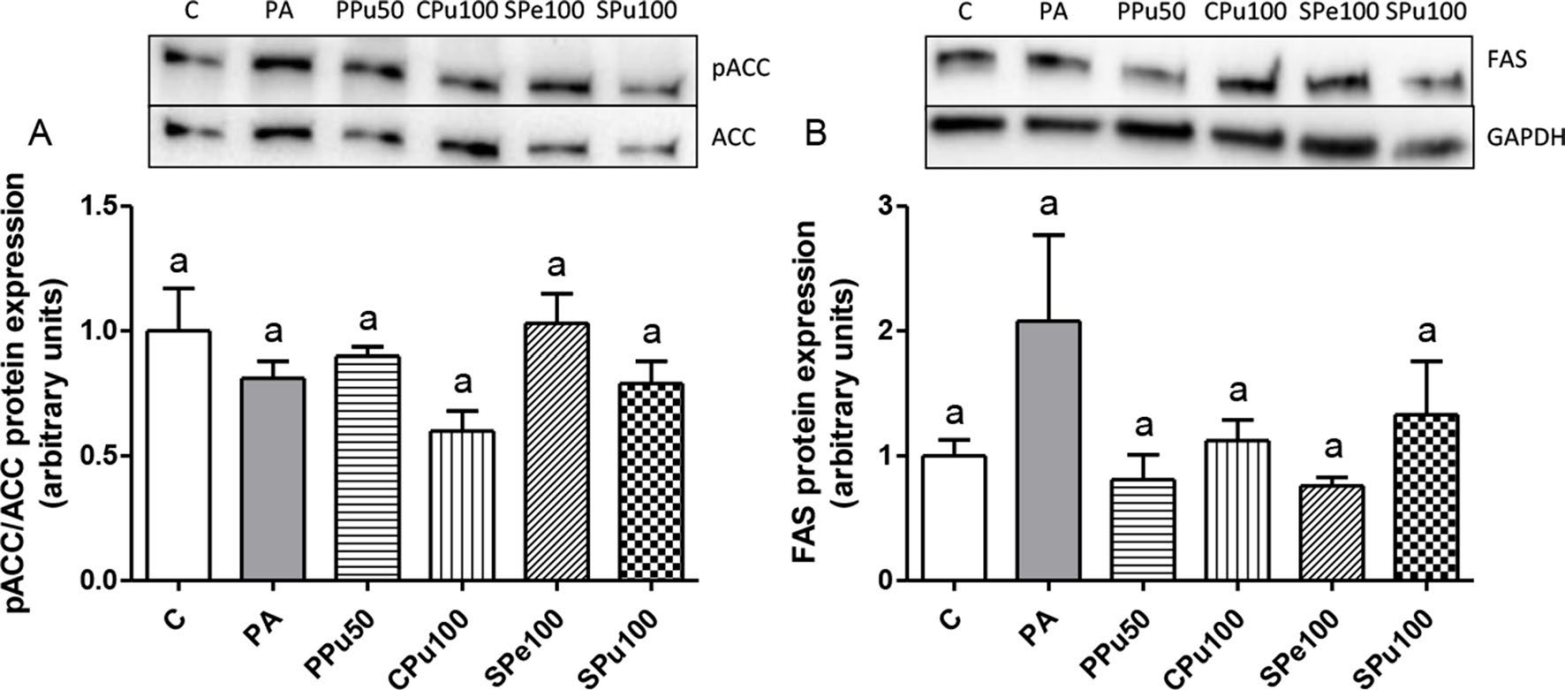
pACC/ACC ratio (**A**) and protein expression levels of FAS (**B**) in AML12 hepatocytes treated with 0.3 mM palmitic acid (PA) in the presence or absence of *Opuntia ficus-indica* extracts: *Pelota* pulp at 50 μg/mL (PPu50), *Colorada* pulp at 100 μg/mL (CPu100), *Sanguinos* peel at 100 μg/mL (SPe100) and *Sanguinos* pulp at 100 μg/mL (SPu100) for 18 h. The Western blots are representative of six samples per group. Data are presented as means ± SEM. Differences among groups were determined using a one-way ANOVA followed by the New-man-Keuls post hoc test. Values not sharing a common letter are significantly different (*p* < 0.05). ACC: acetyl-CoA carboxylase, FAS: fatty acid synthase, GAPDH: glyceraldehyde 3-phosphate dehydrogenase

To identify the mechanism responsible for the lipid-lowering effect of the examined *Opuntia ficus-indica fruit* extracts, the levels of key proteins involved in lipid metabolism were evaluated using immunoblot analysis. For this experiment, PA-treated AML12 hepatocytes were exposed to the extracts and dosages found to induce the highest reductions in triglyceride levels. Since ACC is deactivated through phosphorylation, the pACC/ACC ratio was used as a measure of ACC activity, with a reduced ratio meaning higher enzyme activation. Results showed no significant differences between control and PA groups, nor among cells treated with the different *Opuntia ficus-indica* extracts (Fig. 5A). For FAS protein levels, albeit the difference between the control and PA groups was not statistically significant (*p* = 0.09), a 108% increase was observed in PA hepatocytes. Both PPu50 and SPe100 groups showed reduced values of FAS (61%; *p* = 0.09 and 64%; *p* = 0.08, respectively) when compared to PA cells (Fig. 5B).

**Fig. 6:**
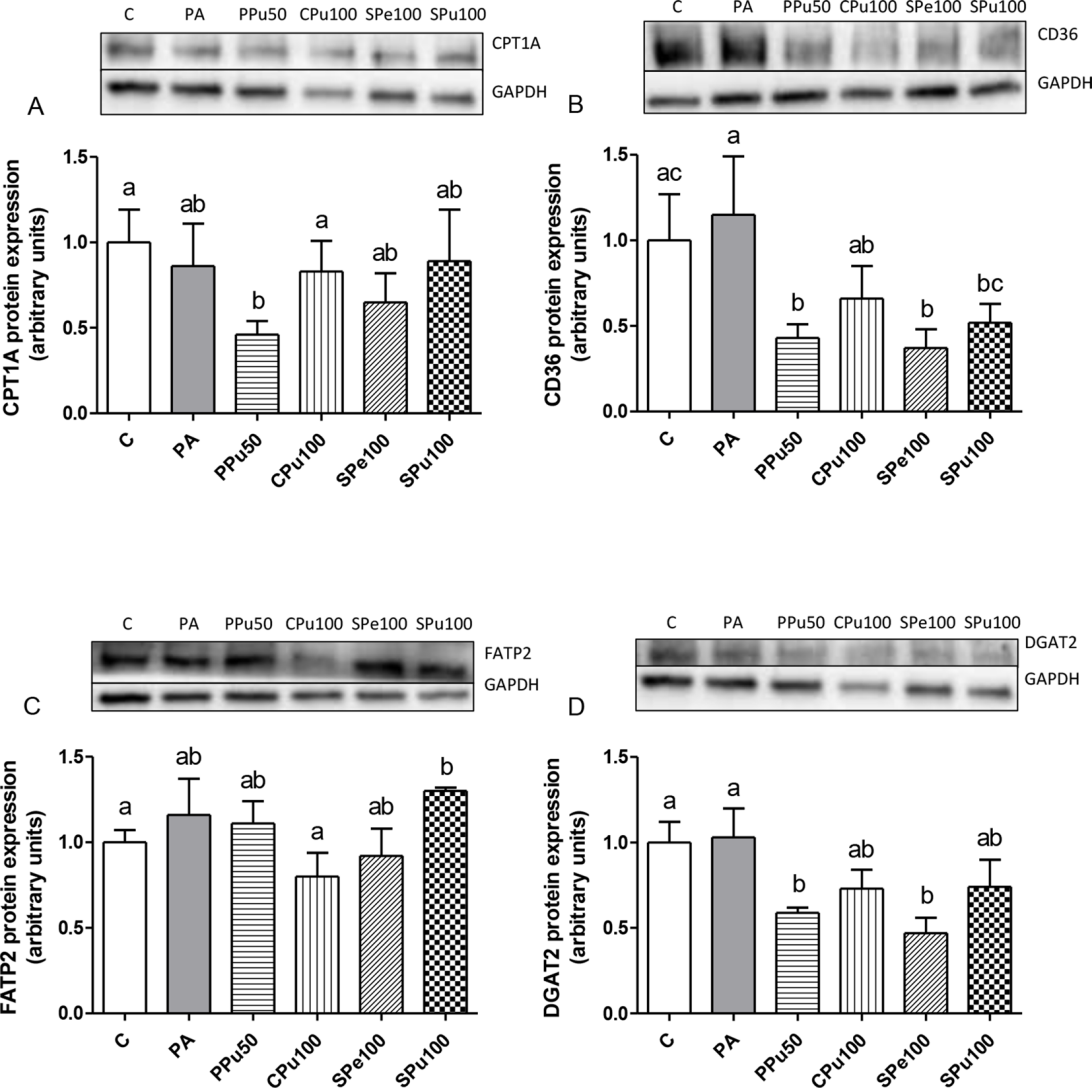
Protein expression levels of CPT1A (**A**), CD36 (**B**), FATP2 (**C**) and DGAT2 (**D**) in AML12 hepatocytes treated with 0.3 mM palmitic acid (PA) in the presence or absence of *Opuntia ficus-indica* extracts: *Pelota* pulp at 50 µg/mL (PPu50), *Colorada* pulp at 100 µg/mL (CPu100), *Sanguinos* peel at 100 µg/mL (SPe100) and *Sanguinos* pulp at 100 µg/mL (SPu100) for 18 h. The Western blots shown are representative of six samples per group. Data are presented as means ± SEM. Differences among groups were determined using one-way ANOVA followed by the New-man-Keuls post hoc test. Values not sharing a common letter are significantly different (*p* < 0.05). CD36: CD36 molecule, CPT1A: carnitine palmitoyltransferase 1 A, DGAT2: diacylglycerol *O*-acyltransferase 2, FATP2: fatty acid transport protein 2, GAPDH: glyceraldehyde 3-phosphate dehydrogenase

Regarding the lipid oxidation pathway, PA exposure did not impact CPT1A protein levels; however, a 46% reduction was noted in the PPu50 group compared to PA-treated hepatocytes (Fig. 6A, *p* = 0.07). Protein expression of the membrane fatty acid transporters CD36 and FATP2 was also analysed, and no significant differences were detected between PA cells and the controls. Concerning *Opuntia ficus-indica* treatments, CD36 levels were significantly reduced in the PPu50, SPe100 and SPu100 groups when compared to PA-treated hepatocytes (Fig. 6B). It is worth noting that all treatments resulted in lower levels of this protein compared to the controls, with a significant reduction observed in the PPu50 and SPe100 groups. FATP2 protein levels were not significantly reduced by any of the *Opuntia ficus-indica* treatments compared to PA group (Fig. 6C). Only a tendency (*p* = 0.1) towards lower values was observed in the CPu100 group relative to PA-treated hepatocytes. DGAT2 protein expression was evaluated to determine triglyceride assembly in hepatocytes, with no significant changes identified following PA treatment alone (Fig. 6D). Cells incubated with PA as well as PPu50 and SPe50 treatments significantly reduced DGAT2 levels compared to the PA group. Moreover, the CPu100 group exhibited a reduction of 29% in DGAT2 expression (*p* = 0.09) compared to the group exposed to PA alone.

### Triglyceride levels in hepatic organoids treated with *Opuntia ficus-indica* extracts

**Fig. 7:**
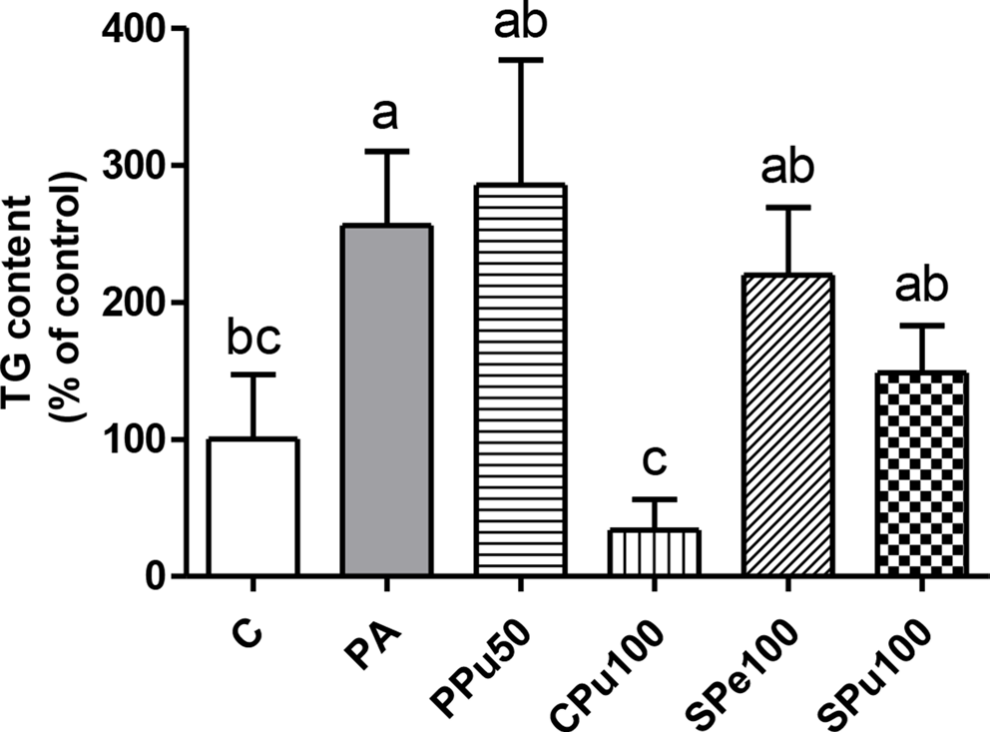
Triglyceride (TG) content in murine hepatic organoids treated with 0.3 mM palmitic acid (PA) in the presence or absence of *Opuntia ficus-indica* extracts: *Pelota* pulp at 50 µg/mL (PPu50), *Colorada* pulp at 100 µg/mL (CPu100), *Sanguinos* peel at 100 µg/mL (SPe100) and *Sanguinos* pulp at 100 µg/mL (SPu100) for 18 h. Data are presented as means ± SEM. Differences among groups were determined using one-way ANOVA followed by the New-man-Keuls post hoc test. Values not sharing a common letter are significantly different (*p* < 0.05)

Hepatic organoids exposed to PA showed significantly higher triglyceride levels compared to the controls (Fig. 7). Co-incubation with PA and the pulp extract of the *Colorada* variety led to a significant reduction in triglycerides compared to the PA group. Moreover, a tendency towards lower levels was observed in organoids treated with the pulp extract of the *Sanguinos* variety (*p* = 0.06).

### Effects of *Opuntia ficus-indica* extracts on genes related to triglyceride metabolism

Triglyceride metabolism-related gene expression was assessed in organoids treated with the doses of each of the four varieties of *Opuntia ficus-indica* extract causing the greatest reduction in triglyceride levels, i.e., 50 µg/mL for the *Pelota* variety pulp extract, and 100 µg/mL for the *Colorada* variety pulp extract, as well as the peel and the pulp extracts of the *Sanguinos* variety.

**Fig. 8:**
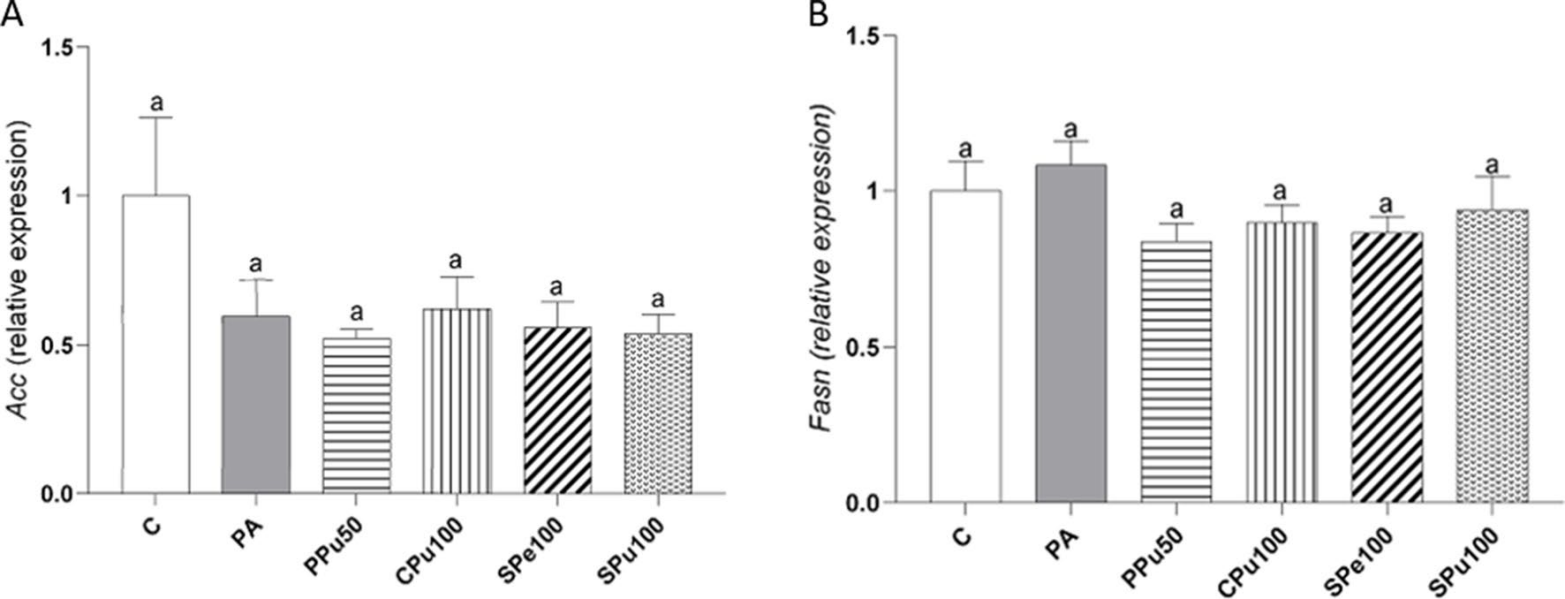
*Acc* (**A**) and *Fasn* (**B**) gene expression in hepatic organoids treated with 0.3 mM palmitic acid (PA) in the presence or absence of *Opuntia ficus-indica* extracts: *Pelota* pulp at 50 µg/mL (PPu50), *Colorada* pulp at 100 µg/mL (CPu100), *Sanguinos* peel at 100 µg/mL (SPe100) and *Sanguinos* pulp at 100 µg/mL (SPu100) for 18 h. Data are presented as means ± SEM. Differences among groups were determined using one-way ANOVA followed by the New-man-Keuls post hoc test. Values not sharing a common letter are significantly different (*p* < 0.05). *Acc*: acetyl-CoA carboxylase, *Fasn*; fatty acid synthase

To further explore on the potential metabolic pathways that lead to triglyceride accumulation in the liver, the expression of genes related to fatty acid synthesis, fatty acid uptake and triglyceride assembly was tested. For *de novo lipogenesis*, gene expressions of *Acc* (Fig. 8A) and *Fasn* (Fig. 8B) remained unchanged in all groups treated with the *Opuntia ficus-indica* extracts.

**Fig. 9:**
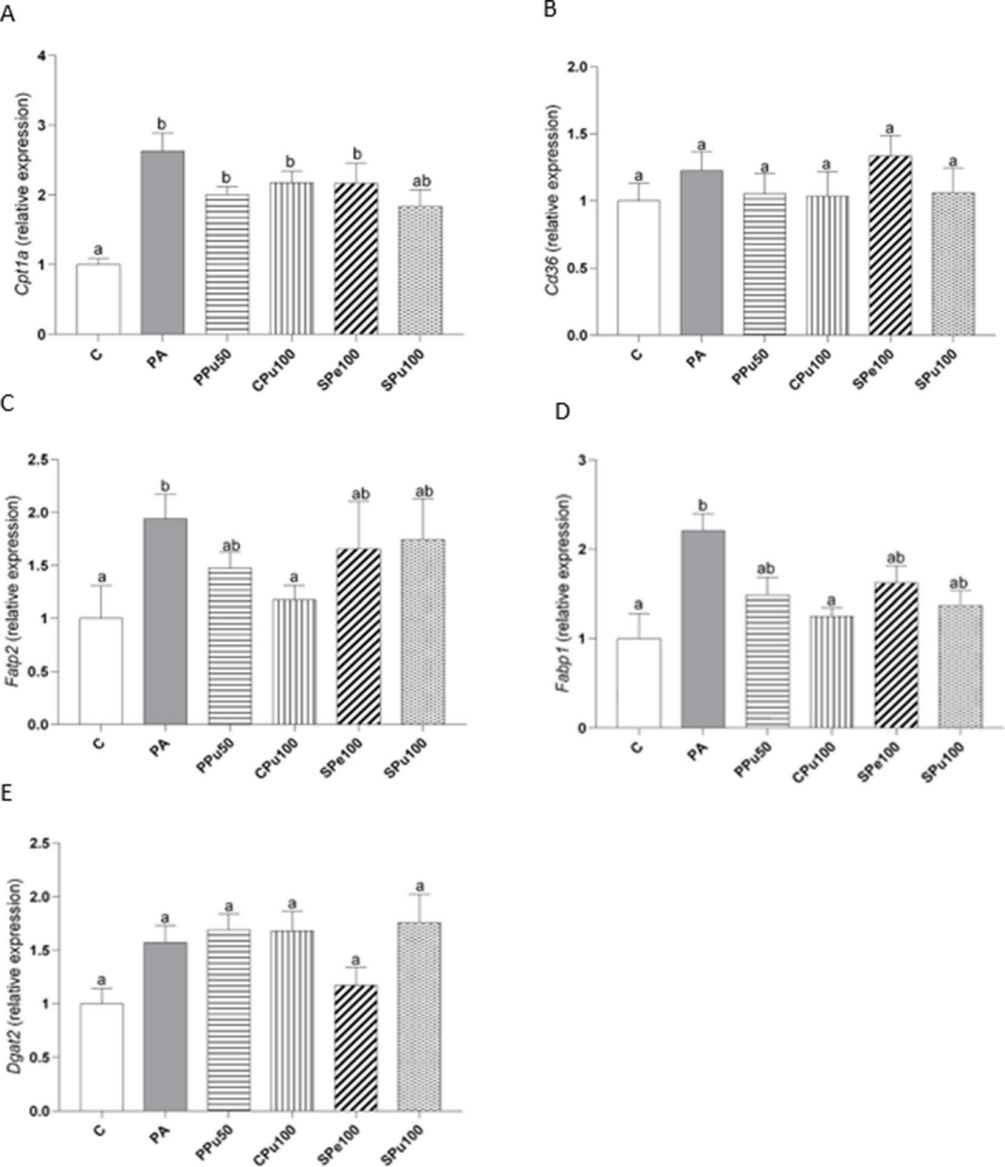
*Cpt1a* (**A**), *Cd36* (**B**), *Fatp2* (**C**), *Fabp1* (**D**) and *Dgat2* (**E**) gene expression in hepatic organoids treated with 0.3 mM palmitic acid (PA) in the presence or absence of *Opuntia ficus-indica* extracts: *Pelota* pulp at 50 µg/mL (PPu50), *Colorada* pulp at 100 µg/mL (CPu100), *Sanguinos* peel at 100 µg/mL (SPe100) and *Sanguinos* pulp at 100 µg/mL (SPu100) for 18 h. Data are presented as means ± SEM. Differences among groups were determined using one-way ANOVA followed by the New-man-Keuls post hoc test. Values not sharing a common letter are significantly different (*p* < 0.05). *Cd36*: CD36 molecule, *Cpt1a*: carnitine palmitoyltransferase 1 A, *Dgat2*: diacylglycerol O-acyltransferase 2, *Fabp1*: Fatty acid binding protein 1, *Fatp2*: fatty acid transport protein 2

With regard to fatty acid oxidation, *Cpt1a* gene expression was analysed. In this case, a significant increase was observed in organoids treated with PA compared to controls, while none of the *Opuntia ficus-indica* treatments induced significant changes (Fig. 9A). Furthermore, the expression of *Cd36* and *Fatp2*, two transporters located in the plasmatic membrane of hepatocytes, and *Fabp1*, a transporter present in the cytosol, were measured to determine fatty acid uptake in the organoids. *Cd36* gene expression was not affected by either PA administration alone or any of the *Opuntia ficus-indica* treatments (Fig. 9B). In contrast, exposure to PA increased the expression of both *Fatp2* and *Fabp1* in hepatic organoids. However, treatment with the pulp extract of the *Colorada* variety completely prevented the PA-induced boost in both genes (Fig. 9C and D). Lastly, mRNA levels of *Dgat2*, which is involved in triglyceride assembly, remained unchanged (Fig. 9E).

## Discussion

It is well known that *Opuntia ficus-indica* extracts exhibit anti-oxidative and anti-inflammatory properties [10, 26]. Given that oxidative stress is a key feature of liver steatosis, and, alongside inflammation, plays an essential role in the progression from fatty liver to more severe conditions such as NASH, it is evident that this plant could represent an interesting tool in the prevention and/or the management of liver steatosis. Nevertheless, before using *Opuntia ficus-indica* extracts for these purposes, it is necessary to determine whether they can reduce hepatic triglyceride accumulation. Limited information on this issue has been published to date. Morán-Ramos et al., (2012) [11] demonstrated that an extract obtained from cladodes, a part of the plant rich in polyphenols (phenolic acids and flavonoids), tocopherols and pectin [5, 6] was able to reduce liver steatosis in genetically obese Zucker rats. Similarly, Sánchez-Tapia et al., (2017) reported the same effect in rats with diet-induced steatosis [13]. Moreover, an extract derived from *Opuntia ficus-indica* seeds, rich in phenolic compounds [12], has been shown to reduce liver triglyceride accumulation. However, to date, no data have been reported on the potential of extracts from fruits or by-products like peels, which, in addition to phenolic compounds [6], contain considerable amounts of betalains.

The fruits of *Opuntia ficus-indica*, also known as prickly pear fruits, can be consumed fresh or used to produce derived products, such as juices and jams. During the preparation of *Opuntia* derived products, a significant amount of waste and by-products is generated, primarily from the fruit peels. Taking into account that prickly pear peels are rich in bioactive compounds, their use as sustainable sources aligns with the principles of a circular economy, offering benefits not only for health but also for the management of bio-wastes, thereby contributing to environmental sustainability [27].

In this scenario, in the present study, both pulp and peel extracts of *Opuntia ficus-indica* were analysed, focusing on the potential beneficial effects in the prevention of liver steatosis. Moreover, given the significant differences in betalain and polyphenol content among various varieties of *Opuntia ficus-indica*, we found it relevant to compare three varieties, *Pelota* (grown in Mexico), and *Colorada* and *Sanguinos* (both grown in Spain). These extracts were used to conduct an in vitro screening using steatotic hepatocytes. Moreover, to our knowledge, this is the first study to analyse the mechanism of action underlying the observed anti-steatotic effect of *Opuntia ficus-indica* extracts and to test them in hepatic organoids.

Hepatocyte incubation revealed that, in general, peel extracts were less effective than pulp extracts. While the pulp concentrate of all three *Opuntia ficus-indica* varieties significantly reduced triglyceride accumulation, only the extract from the *Sanguinos* peel exhibited an anti-steatotic effect. These differences are probably related to the composition of bioactive compounds. The pulp extracts, which were more effective than the peel extracts, contained higher amounts of indicaxanthin (a betalain characteristic of *Opuntia ficus-indica*, but not all *Opuntia* species), lower amounts of piscidic acid, and no detectable isorhamnetin glucosides. The doses with the maximum triglyceride reduction capacity for each extract were 50 µg/mL for the pulp extract of the *Pelota* variety, and 100 µg/mL for the pulp extracts of the *Colorada* and *Sanguinos* varieties, as well as for the peel extract of the *Sanguinos* variety. Comparison of these treatments revealed no statistical differences, indicating that all were equally effective in achieving the maximum triglyceride reduction. In addition, the *Sanguinos* peel extract was the strongest, as it showed an anti-steatotic effect at 25 µg/mL, the lowest dose used in the present experiment, in contrast to other extracts. It should be noted that none of the extracts exhibited a dose-response pattern.

It is noteworthy that the results obtained in the present study differ considerably from those of a previous investigation conducted in our laboratory with *Opuntia stricta* var. *dillenii* fruit extracts (data submitted for publication). In this case, both peel and pulp extracts were more effective, inducing higher triglyceride reductions (17.9–36.9% for peel extracts and 22.3–29.7% for pulp extracts) than those observed in the present study. These differences may again be attributed to variations in bioactive compound composition. Piscidic acid is less abundant in *Opuntia stricta* var. *dillenii* than in *Opuntia ficus-indica*. Moreover, concerning betalains, whereas *Opuntia stricta* var. *dillenii* extracts contain phyllocactin and neobetanin, *Opuntia ficus-indica* extracts contains indicaxanthin. Consequently, the effectiveness of fruit extracts depends on several factors, including the part of the fruit used for extraction and the specific *Opuntia* species.

To elucidate the mechanisms underlying the decreased triglyceride accumulation caused by the *Opuntia ficus-indica* fruit extracts, their effects on key metabolism pathways related to fatty acid and triglyceride metabolism were evaluated. *De novo lipogenesis* is a pathway that results in the biosynthesis of fatty acid chains, which is primarily regulated by ACC and FAS [28]. None of the studied *Opuntia* treatments changed the pACC/ACC ratio or FAS protein expression significantly. Nevertheless, it is important to note that PA incubation of hepatocytes resulted in a 108% increase in FAS expression. Notably, the pulp extract of the *Pelota* variety and the peel extract of the *Sanguinos* variety completely prevented these effects, reducing FAS levels to control values. The other two groups (the pulp extracts of *Colorada* and *Sanguinos* varieties) partially mitigated the effect. These findings suggest that these extracts exert their action by inhibiting the PA-induced increase in *de novo* lipogenesis. Non-esterified fatty acids are absorbed by hepatocytes through transporters like CD36 and FATP2 [29]. Regarding these proteins, when hepatocytes were co-cultured with PA and the pulp extract of the *Pelota* variety, as well as the peel and the pulp extracts of the *Sanguinos* variety, they showed reduced CD36 protein expression. It should be noted that the pulp extract of the *Colorada* variety reduced FATP2 expression to control levels. These results indicate that the *Opuntia ficus-indica* treatments led to a lower fatty acid availability for triglyceride synthesis by limiting uptake from the medium. DGAT2 catalysed the concluding phase of triglyceride synthesis [30]. Both the pulp extract of the *Pelota* variety and the peel extract of the *Sanguinos* variety lessened DGAT2 protein expression, indicating a decrease in triglyceride assembly. In this instance, the pulp extract of the *Colorada* variety exhibited same levels of this protein compared to both controls and PA-treated hepatocytes, although this difference only approached statistical tendency. These findings imply that the reduction in triglyceride levels induced by these *Opuntia ficus-indica* extracts, might be attributed to a combination of decreased fatty acid intake and a reduction in triglyceride assembly.

A second experiment was conducted using murine hepatic organoids, a model that simulates organ and tissue structures, while demonstrating a degree of complex native tissue organisation and function [31], to determine whether the anti-steatotic properties of *Opuntia ficus-indica* extracts were maintained using this methodology. In this case, the pulp extract from the *Colorada* variety at 100 µg/mL was the only treatment able to inhibit triglyceride accumulation induced by PA. Although this may seem surprising, other studies using both AML12 cell line and organoids have reported differing results between the two models. For example, a study by Sun et al., (2019) [32] demonstrated that, after exposure to perfluorooctanoic acid, reactive oxygen species levels were increased in AML12 monolayer cells but decreased in 3D cultures. Moreover, the authors observed a greater reduction in cell viability in AML12 hepatocytes after perfluorooctanoic acid exposure compared to organoids. These findings suggest that organoids are less sensitive to treatments compared to 2D cultures. This is in line with our results, where only the pulp extract of the *Colorada* variety was effective in reducing triglycerides in hepatic organoids, while all four extracts were effective in AML12 hepatocytes.

Upon modifying the culture conditions to promote hepatic maturation as established by Broutier et al. (2016), mouse intrahepatic cholangiocyte organoids exhibited a significant upregulation of mature hepatic markers, including Alb (50-fold increase), Hnf4α (3-fold increase), Cytochrome P450 family 1 subfamily A polypeptide 2 (Cyp1a2; 2-fold increase), Cytochrome P450 family 3 subfamily A polypeptide 11 (Cyp3a11; 2-fold increase), and Glucose-6-phosphatase catalytic (G6pc; 2-fold increase), compared to organoids treated only with expansion media (results not shown), which is in agreement with this validated differentiation methodology. Nonetheless, it should be mentioned that, despite the rigorousness of the protocol used here, organoid hepatic models are not without limitations and results should be interpreted accordingly. Based on our results, the pulp extract of the *Pelota* variety and the peel and pulp extracts of the *Sanguinos* variety are likely ineffective in preventing hepatic steatosis in an in vivo model. Nevertheless, given the limitations mentioned above, further in vivo studies are necessary to confirm the effectiveness of these extracts.

Concerning the mechanisms of action underlying the observed effects, our results indicate that PA exposure to organoids significantly increased *Fabp1*, *Fatp2* gene expression, suggesting enhanced fatty acid uptake. The anti-steatotic effect of the pulp extract of the *Colorada* variety in hepatic organoids appears to result from the downregulation of fatty acid transporters, thereby reducing the supply for triglyceride synthesis. In addition, a significant increase was found in the expression of *Cpt1a*, which allows long-chain fatty acids to enter the mitochondria for oxidation [33]. This effect may represent a compensatory mechanism aimed at preventing excessive triglyceride accumulation, as observed in in vivo studies [34].

The results obtained in the present study support a potential role for *Opuntia ficus-indica* extracts as valuable tools for liver steatosis prevention. Nevertheless, further research is needed to assess the bioavailability of the bioactive compounds. To date, studies in humans have shown that after the ingestion of cactus pear pulp, the maximum plasma concentration of indicaxanthin and betanin occurred 3 h after ingestion [10, 35, 36], with concentrations of 1.03 ± 0.2 µM and 30 ± 5.2 nM, respectively. It has been also observed that the half-life of indicaxanthin is much longer than that of betanin [10, 35]. In this context, it should be noted that we conducted a study to analyse the bioaccesibility of the most abundant betalains and phenolic compounds in *Opuntia stricta* var. *dillenii* whole fruit, peel, pulp, bagasse, jam and fresh pressed juice, using the INFOGEST^®^ methodology. Although the results revealed significant differences depending on the compound class and the type of product, considerable losses in both phenolic compounds and betalains were generally observed [37]. Similar findings have been reported by other authors when analysing extracts of *Opuntia ficus-indica* [10]. Regarding phase II metabolism of these compounds, limited information is available [35, 38].

## Conclusions

In summary, the screening conducted in AML12 hepatocytes shows that the fruits of *Opuntia ficus-indica* could serve as promising materials to produce extracts with anti-steatotic activity. Among the three varieties studied, *Sanguinos* appears to be the most remarkable, since both pulp and peel extracts exhibited activity. This is an important aspect, as there is a growing need to develop new strategies for managing agricultural food processing wastes and residues, given that food by-products exceed a billion tons generated annually. For the pulp extracts of *Pelota* and *Colorada* varieties, and the peel extract of *Sanguinos*, it is proposed that the mechanism underlying the anti-steatotic effect involves the downregulation of fatty acid uptake and triglyceride assembly. In the case of the pulp extract of *Sanguinos*, the effect would be caused solely by reduced fatty acid transport. However, when testing these extracts in hepatic organoids, only the pulp extract of the *Colorada* variety was effective in preventing triglyceride accumulation, an effect mediated by reduced fatty acid uptake. As previously described in the present paper, organoids better replicate the actual in vivo physiology and functions of the hepatic tissue compared to traditional 2D monocultures. Considering this and the present results, it is likely that the Colorada extract holds a higher probability of actually being effective in vivo. Nevertheless, further studies are needed to confirm this hypothesis.

## Data Availability

No datasets were generated or analysed during the current study.
